# Supplementary material for: A Rare Nestin-Expressing Granule Cell Precursor subpopulation Underlies SHH Medulloblastoma Formation
Source: bioRxiv. 2026 May 26:2026.05.26.727818. Preprint. [Version 1] doi: 10.64898/2026.05.26.727818 (PMC13232223; doi:10.64898/2026.05.26.727818)
Supplement: 1 [file NIHPP2026.05.26.727818v1-supplement-1.pdf]

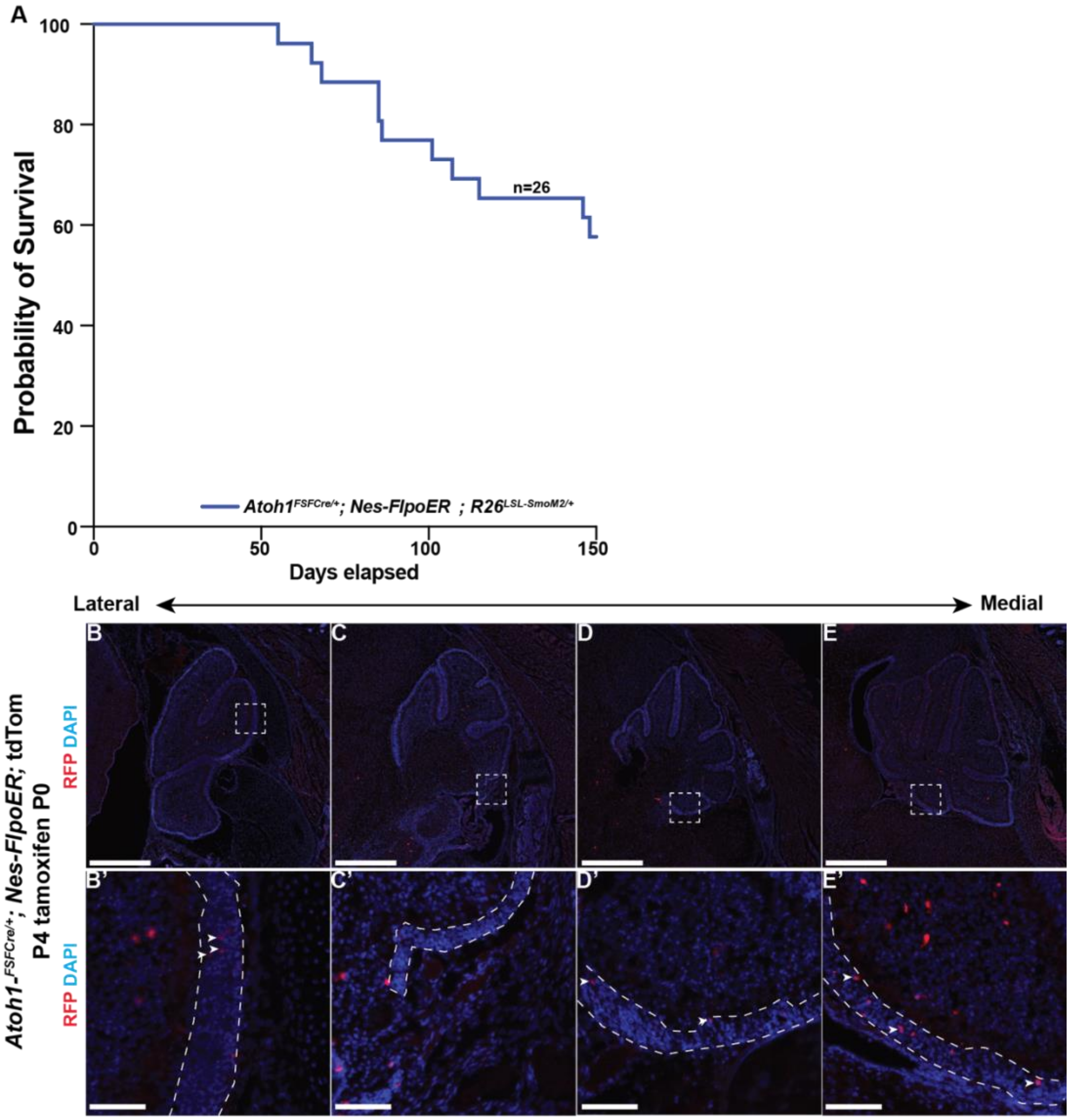

Figure 4 – figure supplement 1. A dual recombinase strategy confirms SHH MB tumors can initiate from *Nes*-expressing GCPs (*Atoh1*$^{+}$).

(A) Kaplan-Meier survival curve of *Atoh1*$^{Frt\text{-}Cre/+}$*; Nes-FlpoER; R26*$^{LSL\text{-}SsmoM2/+}$ mice following tamoxifen induction at P0.

(B-E) Immunofluorescence staining of sagittal cerebellar sections from *Atoh1*$^{Frt\text{-}Cre/+}$*; Nes-FlpoER;* tdTom mice collected at P4 following tamoxifen injection at P0 showing RFP (red) and DAPI (blue). Sections are shown at four distinct lateral-medial levels. (B'-E') High magnification images of boxed regions in Panels (B-E). White arrowheads indicate the tdTomato^{+} cells (RFP) in the EGL. N>3 mouse samples.

Scale bars: B-E: 500um, B’-E’: 50um

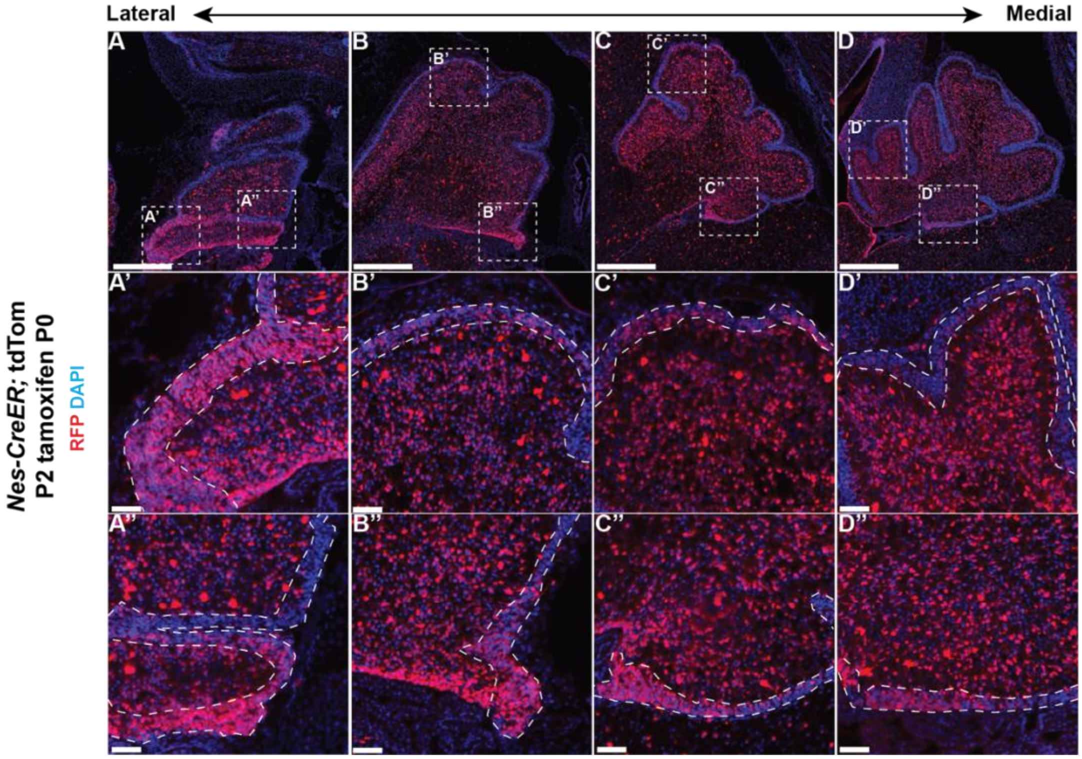

Figure 4 – figure supplement 2. The *Nes-CreER* line labels a much broader progenitor population of cells than the *Nes-FlpoER* line.

(A-D) Immunofluorescence staining of sagittal cerebellar sections from *Nes-CreER;* tdTom mice (*Nes-CreER; R26*$^{LSL\text{-}tdTom}$) collected at P2 following tamoxifen injection at P0, showing RFP (red) and DAPI (blue). Sections are shown at four distinct lateral-medial levels. (A'-D') High magnification images of anterior cerebellum. (A''-D'') High magnification images of posterior cerebellum. N= 3 mice.

Scale bars: A-D: 500um and A'-D', A''-D'': um

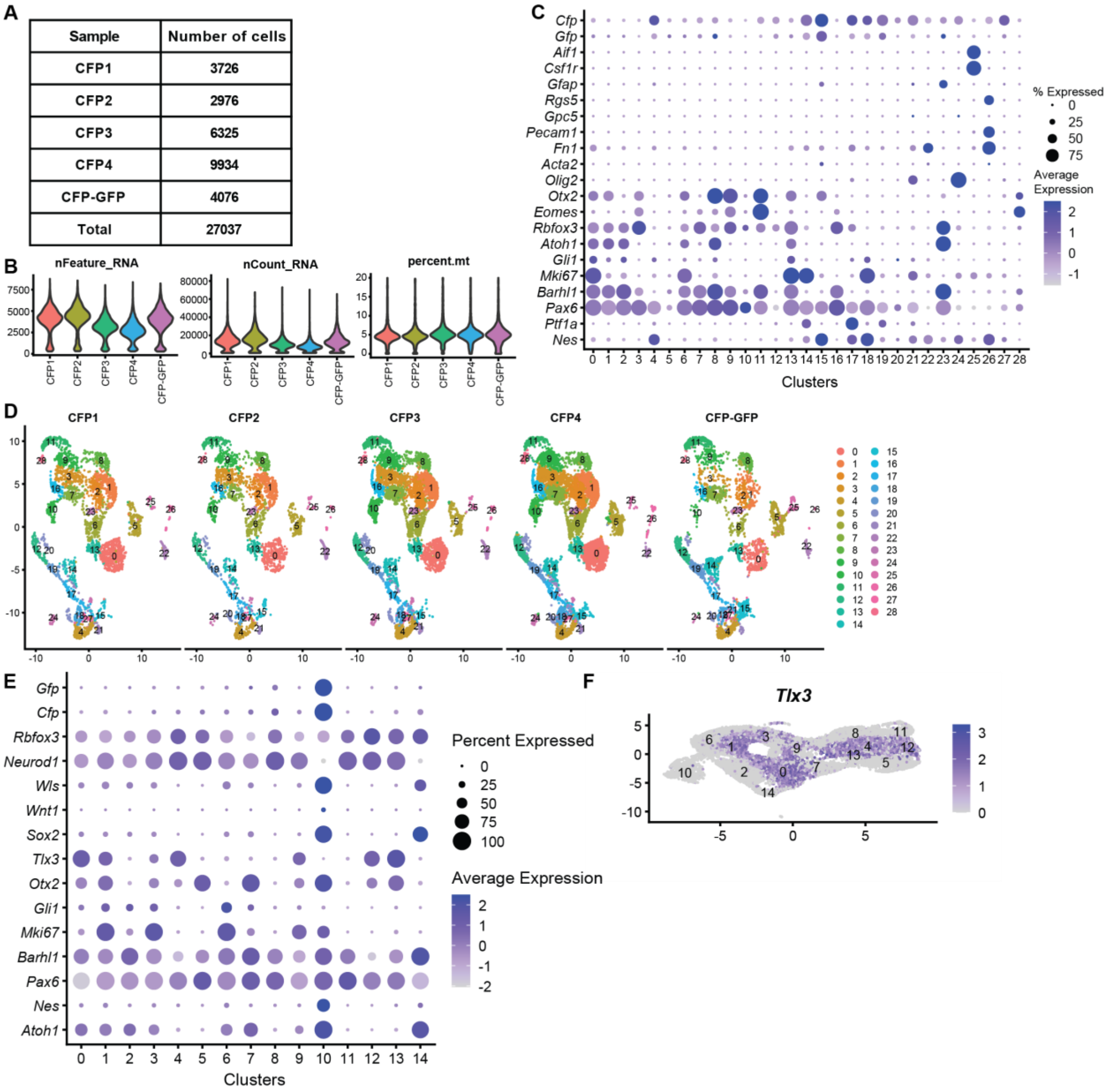

Figure 5 – figure supplement 1. ScRNA-seq workflow and additional characterization of the *Nes*-expressing GCP clusters.

(A) Number of cells from each sample used for downstream analyses after filtering. Samples CFP1 to CFP4 represent 1 animal each and CFP-GFP represent 3 animals pooled.

(B) Violin plots showing the number of features, the RNA counts and the percent of mitochondrial RNA counts in each sample.

(C) Dot plot graph showing expression levels of cell type marker genes across all clusters for all cells. See also Figure 5-Source data 1.

(D) UMAPs of all cells shown by samples. CFP1 to CFP4 are the posterior GCP samples from *Nes-CFP* mice (one mouse per sample) at P1 and CFP-GFP corresponds to the sample from *Atoh1-GFP*; *Nes-CFP* mice where CFP^{+} GFP^{+} cells were isolated by FACS (n=3 mice combined).

(E) Dot plot graph showing expression levels of GCP markers across the integrated GCP dataset. See also Figure 5-Source data 2.

(F) Feature plots showing expression of posterior marker *Tlx3* in GCP clusters in the integrated GCP dataset. See also Figure 5-Source data 2.
